# Cytoplasm-Translocated Ku70/80 Complex Sensing of HBV DNA Induces Hepatitis-Associated Chemokine Secretion

**DOI:** 10.3389/fimmu.2016.00569

**Published:** 2016-12-05

**Authors:** Young Li, Yang Wu, Xiaohu Zheng, Jingjing Cong, Yanyan Liu, Jiabin Li, Rui Sun, Zhigang G. Tian, Haiming M. Wei

**Affiliations:** ^1^The CAS Key Laboratory of Innate Immunity and Chronic Disease, Institute of Immunology, School of Life Sciences, University of Science and Technology of China, Hefei, China; ^2^University of Science and Technology of China, Hefei, China; ^3^Department of Infectious Diseases, The First Affiliated Hospital of Anhui Medical University, Hefei, China; ^4^Hefei National Laboratory for Physical Sciences at Microscale, Hefei, China

**Keywords:** HBV, DNA sensor, chemokine, innate immunity, IRF1

## Abstract

Chronic hepatitis B virus (HBV) infection remains a serious disease, mainly due to its severe pathological consequences, which are difficult to cure using current therapies. When the immune system responds to hepatocytes experiencing rapid HBV replication, effector cells (such as HBV-specific CD8+ T cells, NK cells, NKT cells, and other subtypes of immune cells) infiltrate the liver and cause hepatitis. However, the precise recruitment of these cells remains unclear. In the present study, we found that the cytoplasm-translocated Ku70/80 complex in liver-derived cells sensed cytosolic HBV DNA and promoted hepatitis-associated chemokine secretion. Upon sensing HBV DNA, DNA-dependent protein kinase catalytic subunit and PARP1 were assembled. Then, IRF1 was activated and translocated into the nucleus, which upregulated CCL3 and CCL5 expression. Because CCR5, a major chemokine receptor for CCL3 and CCL5, is known to be critical in hepatitis B, Ku70/80 sensing of HBV DNA likely plays a critical role in immune cell recruitment in response to HBV infection.

## Introduction

According to data from the World Health Organization, hepatitis B virus (HBV) infection is an ongoing major health concern worldwide, with an estimated 240 million people chronically infected. Moreover, HBV-associated complications, including cirrhosis and liver cancer, kill more than 780,000 people worldwide annually (1). Unfortunately, the major HBV therapies currently available, such as interferon and/or nucleotide analog treatments, are unsatisfactory in terms of virus eradication (2). Although much knowledge regarding this enigmatic disease remains unclear, researchers have identified some common immune responses in hepatitis B. For example, various immune cell types play a role in the development of acute and chronic hepatitis, including NK, CD4+ T, and CD8+ T cells (3). These cells accumulate at the site of infection and contribute to the process of hepatitis, resulting in hepatocyte damage and an increase in serum alanine aminotransferase (ALT). However, the way in which the recruitment of immune cells is initiated requires further elucidation. Many studies have revealed the crucial roles of cytokines during viral hepatitis progression, and these include immune cell activation and suppression (4–6), viral clearance (7, 8), and effector cell recruitment (9–11). Determining how the first round of cytokine secretion occurs, which leads to the resulting cascade of immune reactions, is thus essential to understand the mechanisms underlying viral hepatitis.

The innate immune system serves as the vanguard in host defense, before the adaptive immune system responds. Pattern-recognition receptors (PRRs) recognize pathogen-associated molecular patterns (PAMPs) at the front line of the interaction between host and pathogen and subsequently engage various pathways that lead to host-protective mechanisms (12). As all microbes contain DNA and/or RNA, nucleic acids constitute an important type of PAMP, especially in viral infection. Recently, numerous studies on cytosolic nuclear acid-sensing pathways have identified the key roles of cytosolic nuclear acid sensors in protecting the host against invading pathogens (13). Regarding HBV, a DNA virus with a special life cycle that involves reverse-transcribed genomic DNA, both viral RNA and DNA, are present in the host cell cytoplasm (14–16). RIG-I has been shown to be responsible for HBV pre-genomic RNA (pgRNA) sensing and antiviral activity in HBV-expressing cells (17). In human immunodeficiency virus (HIV) infection and other retrovirus infections, cyclic GMP-AMP synthase (cGAS) acts as an important sensor of cDNA (18–20). The DNA sensor IFI16 has also been shown to be involved in the response to viral infection (21, 22). Considering the similar reverse transcription replication strategy of HBV and retroviruses, it seems possible that similar DNA-sensing pathways exist in HBV infection. For instance, cGAS has been reported to be required for the innate immune response against HBV (23). However, much is unknown about HBV DNA sensing.

In this study, we observed that chemokines, especially CCL5, are expressed at very high levels in patients with HBV-associated disease, by using data mining of the Gene Expression Omnibus (GEO). Serum test results of HBV patients and Ad-HBV infection of mice also confirmed this observation. CCL5 and CCL3 are well characterized as inflammation-associated chemokines (24–26), and their main receptor, CCR5, is also essential in hepatitis B progression (27). We found that the Ku70/80 complex senses HBV DNA in the cytoplasm and upregulates CCL3 and CCL5 expression in an HBV DNA transfection model. The Ku70/80 complex is well known to be a DNA damage repair factor that monitors DNA breaks and non-homologous end joining (NHEJ) in the nucleus (28). Although Ku protein translocation to the cytoplasm has been reported about a decade ago (29, 30), it was not until recently that Ku’s DNA sensor capability was identified (31, 32). Zhang et al. revealed that Ku70 senses DNA in different forms and promotes type III interferon expression in an IRF1/IRF7-dependent manner. In addition, data reported by Ferguson et al. showed that Ku proteins sense viral DNA together with the DNA-dependent protein kinase catalytic subunit (DNA-PKcs) and that signal transduction involves TBK1–IRF3. Moreover, when the DNA-binding capability of Ku70/80 complex is blocked by vaccinia virus component C16, cytokines, such as IL-6, CXCL10, induced by DNA stimulation is downregulated (33). The data reported in this study identify a different mechanism by which the Ku70/80 complex coordinates with DNA-PKcs and PARP1 to activate an IRF1-dependent pathway and upregulate CCL3 and CCL5 chemokine expression upon sensing HBV DNA in the cytoplasm. In Ad-HBV-infected mice, CCL3 and CCL5 contributed to the recruitment of effector immune cells to the liver and promoted hepatitis. Moreover, knockdown of Ku70 expression significantly affected CCL3 and CCL5 secretion. Considering the critical role of CCR5 in hepatitis B (27), this type of DNA-sensing-dependent chemokine upregulation mechanism may explain how immune cells are recruited in response to HBV infection.

## Materials and Methods

### GEO Dataset Analysis

Gene expression profile data (GSE38941 and GSE65359) were downloaded from GEO: https://www.ncbi.nlm.nih.gov/geo (34). Student’s *t*-tests, hierarchical clustering, and heat map analysis were performed using MultiExperiment Viewer (MeV) (35). Gene set enrichment analysis (GSEA) was performed using the GSEA software (36).

### HBV-Infected Patients

Twenty patients with chronic hepatitis B were selected randomly from the cohort studied in our lab previously (37). The patients received continuous antiviral treatment of 1.5 μg/kg Peg-IFNα-2b (PegIntron, Schering-Plough, Kenilworth, NJ, USA) weekly or Peg-IFNα-2b weekly combined with 10 mg adefovir dipivoxil (ADV, Hepsera, Gilead Sciences, Foster City, CA, USA) daily for 48 weeks. Patient sera collected at 0 and 48 weeks were tested for CCL5 concentration by BD Cytometric Bead Array.

All serum samples of HBV patients were collected at the Department of Infectious Diseases, the First Affiliated Hospital of Anhui Medical University from June 2012 to July 2014. Written informed consent was obtained from all patients donating blood samples. The study was approved by the ethics committee of the First Affiliated Hospital of Anhui Medical University (Grant No. K2010003) and was carried out in accordance with the approved guidelines. This clinical research was also enrolled in the Chinese Clinical Trial Registry (Clinical trial registration number: ChiCTR-TRC-12002226). The characteristics of the HBV patients are listed in Table 1.

**Table 1:** **Characteristics of HBV patients**.

Characteristics	Pre-treatment	Treatment for 48 weeks
HBsAg (SD), IU/ml	29,945 (43,519)	6107 (8084)
HBeAg (SD), COI	676 (434)	212 (368)
Cases	20	
Male	13	
Mean age (SD)	30.7 (7.5)	
HBV genotype	B(9), C(11)	

### Cell Culture

The cell lines SK-Hep-1 and Huh 7 were provided by the Cell Bank of the Chinese Academy of Sciences. The cell lines HepG2, HepG 2.2.15, and 293A were maintained in our laboratory. The cells were cultured in DMEM containing 10% FBS (Sigma or Gibco) and antibiotics (100 U/ml penicillin, 80 μg/ml streptomycin). Human sinusoidal endothelium cells (HSECs) were purchased from Sciencell. The HSECs were cultured with Endothelial Cell Medium (Sciencell) at 37°C with 5% CO_2_, and the culture medium was renewed every other day.

### Plasmid Construction

The pAAV-HBV1.2 plasmid was provided by Pei-Jer Chen (National Taiwan University, Taipei) and maintained in our laboratory. The pAAV plasmid was subcloned from pAAV-HBV1.2.

The HBV subtype adw sequence was subcloned from the rAAV-HBV1.3 virus (FivePlus Molecular Medicine Institute, Beijing, China). The pAAV-HBV1.3 plasmid was constructed by ligating 1.3 copies of the HBV sequence (subtype “adw,” *Nsi*I + *Eco*RI-cleaved fragment, and *Eco*RI + *Bgl*II-cleaved fragment) into the pAAV plasmid. The pAAV-HBV1.3 plasmid was digested using *Nsi*I, and the HBV sequence fragment was cleaved off and then ligated into the *Nsi*I-linearized pAAV-HBV1.3 plasmid to construct the pAAV-HBV2.3 plasmid.

CCL3 promoter (2 kbp upstream CCL3) was cloned and inserted to replace the CMV promoter of pEGFP plasmid. Then, luciferase reporter sequence was cloned downstream the CCL3 promoter to construct pCCL3p-luc-eGFP plasmid.

### Cell Transfection

Plasmid transfection of SK-Hep-1, Huh 7, and HepG 2 cells was performed using Lipofectamine 2000 or Lipofectamine 3000 (Invitrogen). HSECs were transfected with FuGENE HD (Promega). siRNAs were synthesized by GenPharma, Shanghai, China, and then transfected into cells with RNAiMAX (Invitrogen). Cells were plated 24 h before transfection. All of the transfection experiments were performed according to the manufacturer’s instructions. Briefly, 1 μg/ml plasmid was transfected using 4 μl Lipofectamine 2000/3000 or 2 μl FuGENE HD. The 20 pmol siRNA was transfected with 4 μl RNAiMAX in 24-well plate. Fresh culture medium with 10% FBS was added 24 h post-transfection.

### Luciferase Reporter Assay

SK-Hep-1 cells were transfected with pCCL3p-luc-eGFP plasmid and selected with G418 (Gibco) for stable expression. Then, the cells were challenged with different stimulants. Forty-eight hours later, 150 μg/ml of firefly luciferin was added to the cell culture supernatant and bioluminescence was determined immediately.

### Determination of Chemokine Levels by Enzyme-Linked Immunosorbent Assay

Cell culture supernatant was collected and centrifuged at 16,000 *g* for 10 min to remove cell fragments, and serum samples were prepared by spinning coagulated blood and collecting the supernatant. Tissue samples were grinded with cell lysis buffer on ice centrifuged at 16,000 *g* for 10 min, and then the supernatant was collected. CCL3 and CCL5 enzyme-linked immunosorbent assay (ELISA) kits were purchased from R&D. Experiments were carried out according to the protocols of the manufacturer’s instructions.

### HBV DNA Labeling, Pulldown, and Mass Spectrometry

Biotin-labeled HBV DNA was amplified by PCR with the 5′-biotin sense primer and anti-sense primer from the pAAV-HBV2.3 plasmid, excised from the agarose gel, and then transfected into SK-Hep-1 cells for 6 h. Then, the cells were lysed with NP-40 lysis buffer (150 mM NaCl, 50 mM Tris–HCl, pH = 7.4, 1 mM freshly added PMSF). The DNA-binding proteins were pulled down with a μMACS Streptavidin kit (Miltenyi Biotec). Mass spectrometry results were obtained by APTbiotech, Shanghai, China. Briefly, protein samples were digested with trypsin for 20 h. Then, protein peptides were identified by liquid chromatography (Zorbax 300SB-C18; Agilent) and tandem mass spectrometry (Thermo Finnigan). The mass spectral data were searched against UniProt human proteomic database using the Mascot 2.2 software.

### Mouse Protocols

C57BL/6 mice were purchased from Vitalriver, Beijing, China. All mice were kept in a specific pathogen-free (SPF) microenvironment, receiving care in compliance with the guidelines set forth in the *Guide for the Care and Use of Laboratory Animals*. All experiments were approved by the Ethical Review Board of the School of Life Science, University of Science and Technology of China. Mice aged between 6 and 8 weeks were used for these experiments. For virus infection, the plasmid of recombinant adenovirus that transfers a 1.3-fold overlength HBV genome (Ad-HBV) was provided by Prof. Ulrike Protzer (Technischen Universität München). Ad-null virus was purchased from the FivePlus Molecular Medicine Institute. The virus was packaged and amplified in 293A cells, and the titer was determined by plaque formation. A total of 1 × 10^9^ pfu/200 μl virus was injected intravenously (i.v.) into each mouse. Six days later, the mice were sacrificed.

### Hepatic Mononuclear Cell Isolation and Flow Cytometry

Livers were ground to pass through a 200-gauge stainless steel mesh. The precipitate was re-suspended in 40% Percoll (Sigma) and centrifuged at 2400 rpm for 10 min at room temperature. Then, the precipitate was collected and re-suspended in RBC Lysis Buffer (BioLegend), incubated at room temperature for 10–15 min, and then washed twice in PBS. Splenocytes were obtained in the same manner, except for Percoll re-suspension. Flow cytometry was performed, as previously described (38).

### BD Cytometric Bead Array

Samples were diluted according to the manufacturer’s instructions if necessary. Then, incubated with the mixed beads at 4°C for 30 min, washed the beads with PBS twice, and applied them on flow cytometry.

### Serum Transaminase Assay

Serum ALT activity was measured using a commercially available kit (Rong Sheng, Shanghai, China).

### Immunofluorescence Staining

Cells or frozen tissue sections were fixed in 4% paraformaldehyde (PFA) for 15–20 min, followed by permeabilization with 1% Triton X-100 for 15–20 min, and blocking with 5% donkey serum for 1 h at room temperature. Then, the sections were incubated with the primary antibodies at 4°C overnight. Secondary antibodies were incubated with the sections at 37°C for 3 h. The nuclei were stained with DAPI (1 ng/ml) for 3 min at room temperature. Confocal images were acquired on a Zeiss LS710 microscope, and GSD super-resolution images were acquired using the Leica 3D GSD system.

### mRNA Isolation, Reverse Transcription PCR, and Real-time PCR

Cell or tissue mRNA was isolated with TRIzol and reverse transcribed into cDNA with M-MLV (Invitrogen). Relative quantitative real-time PCR analysis was performed on Roche LightCycler 96 using the SYBR Premix Ex Taq II (Takara). These data were analyzed using ΔΔ*C*_t_ method.

### Cytosolic and Nuclear Protein Extraction

Cells were lysed on ice for 30 min with cytoplasm extraction buffer (10 mM HEPES, 40nM KCl, 2mM MgCl_2_, 10% glycerol, and 1mM PMSF). The cells were lysed on ice for 30 min with cytoplasm extraction buffer (10 mM HEPES, 40 nM KCl, 2 mM MgCl_2_, 10% glycerol, and 1 mM PMSF). The cells were centrifuged at 2000 rpm for 5 min at 4°C, and the supernatant was collected. The supernatant was then centrifuged at 14,000 rpm for 15 min at 4°C. The supernatant was the cytosolic protein solution, and the pellet was used as the nuclear protein sample.

### Co-Immunoprecipitation

Cells were lysed with 500 μl NP-40 lysis buffer, and then centrifuged at 14,000 rpm for 15 min at 4°C. The supernatant was collected, added in 30 μl protein G-Agarose beads (Sangon, Shanghai) together with 1–10 μg isotype IgG antibody, and incubated at 4°C for 1 h with gentle agitation. Then, centrifuged and the beads were discarded, the supernatant added in 30 μl protein G-Agarose beads and 1–10 μg intended antibody. It was incubated at 4°C overnight with gentle agitation. Centrifuged to collect down the beads and washed three times in lysis buffer.

### Western Blot

Protein samples were applied to SDS-PAGE, and then transferred to PVDF membrane (0.45 μm, Millipore). It was incubated with the primary antibody at 4°C overnight or 3 h at room temperature. The membrane was washed in TBST for three times, 5 min each, followed by incubation of HRP-conjugated secondary antibody at room temperature for 1 h. The membrane was washed four times in TBST, and then developed it with SuperSignal West Femto Maximum Sensitivity Substrate (Thermo Fisher).

### Antibodies

Ku70, Ku80, PARP1, IRF1, cGAS, STING primary antibodies, anti-HBsAg, and anti-HBcAg were purchased from Abcam. IRF3, IRF7, RNA pol III, CCL3, and CCL5 antibodies were purchased from Santa Cruz. Secondary antibodies labeled with Alexa Flour were all purchased from Invitrogen. Mouse antibodies were all purchased from BD Bioscience, except for CD8 primary antibody from eBioscience. Refer to Table S1 in Supplementary Material for detailed information.

### PCR Primers and siRNA Sequences

For detailed information on PCR primers and siRNA sequences, see Table S1 in Supplementary Material.

### Statistical Analyses

We used Student’s *t*-tests to compare the mean values between groups. Bars represent the SD of each group (*p < 0.033, **p < 0.002, ***p < 0.0002, and ****p < 0.0001).

## Results

### Chemokines CCL3 and CCL5 Are Involved in Hepatitis B

**Figure 1:**
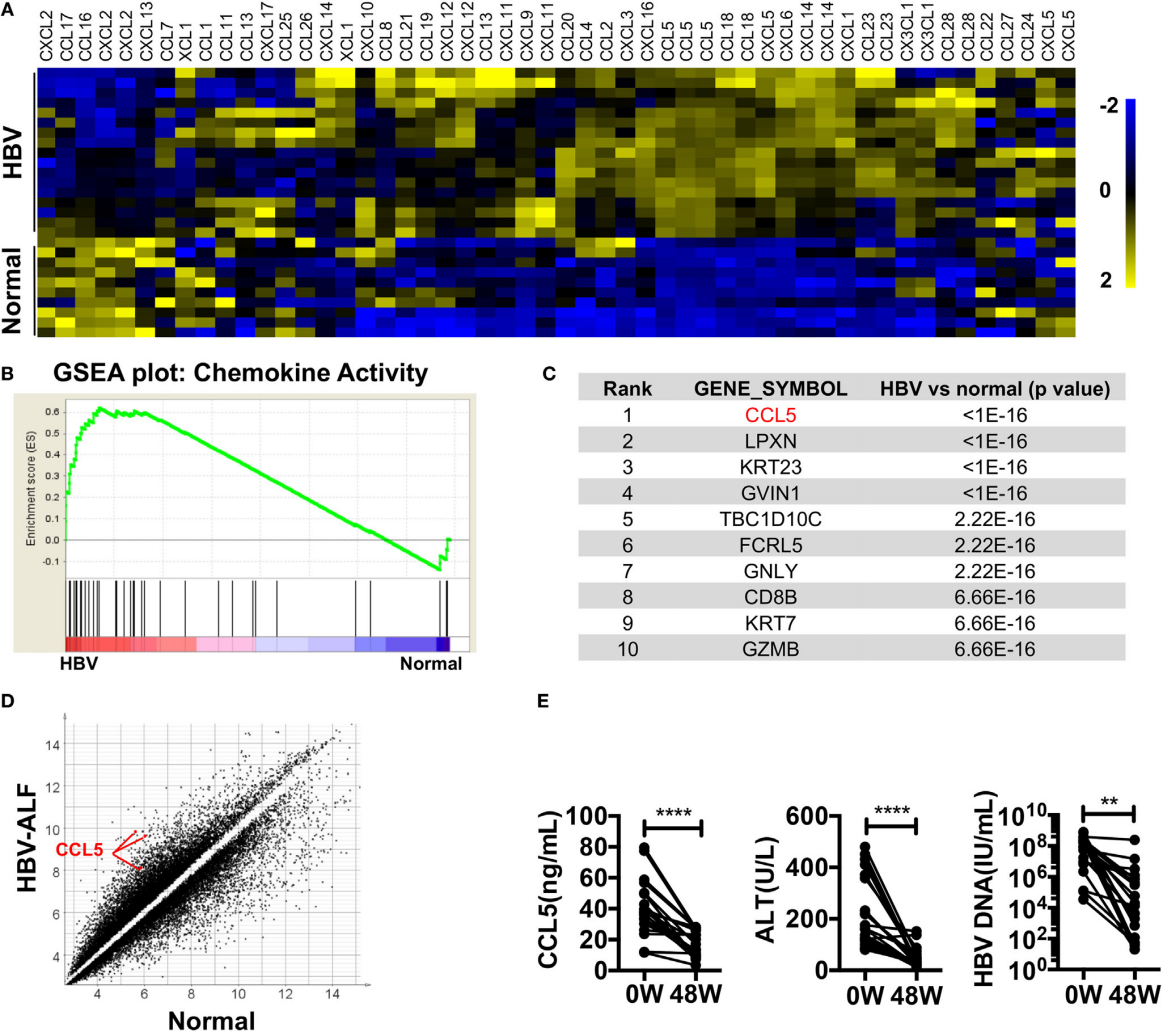
**Chemokines, especially CCL5, are highly expressed in the livers of patients with HBV-associated disease**. **(A)** Heat map of chemokine expression in GSE38941. These data were normalized with MeV. **(B)** GSEA of the chemokine activity gene set in GSE38941. **(C)** Genes with top-ranking *p*-values from Student’s *t*-tests comparing the expression profiles of liver samples from HBV-associated ALF patients with those of healthy donors. These data were derived from GSE38941. **(D)** Scatter plot of genes with *p*-values less than 0.01 in **(A)**. Three CCL5 probes are indicated. **(E)** The levels of CCL5, ALT, and HBV DNA were measured in the serum of patients with hepatitis B. 0 W, serum samples before antiviral treatment; 48 W, serum samples after 48 weeks of antiviral treatment (*n* = 20).

To identify HBV infection-related factors, we searched HBV-associated gene expression profiles in GEO. In dataset GSE38941, which consisted of liver specimens from patients with HBV-associated acute liver failure (ALF) and healthy donors (39), we noticed that most of the chemokines were significantly upregulated in the livers of patients compared with healthy donors (Figure 1A). A GSEA plot of the chemokine activity gene set also showed a strong enrichment of chemokines in the livers of patients (Figure 1B). In particular, CCL5 had a top-ranked *p*-value among genes that were significantly different between patients and donors (Figure 1C). The absolute expression of CCL5 in HBV-associated ALF patients was also much higher than that in healthy donors (Figure 1D). In another dataset, GSE65359 (40), which consisted of which consisted of liver specimens from HBV-infected patients in the immune tolerance, immune clearance, and chronic phases, some chemokines were also highly expressed in patients in the immune clearance phase (Figure S1A in Supplementary Material). Of these chemokines, CCL5 showed a very significant difference and high expression levels in immune clearance group (Figures S1B,C in Supplementary Material). Considering the symptoms of hepatitis in patients with HBV-associated ALF and in patients of immune clearance phase, these data suggest that CCL5 is important in hepatitis B. Next, we tested the serum of HBV patients receiving continuous antiviral treatment (Table 1). As expected, when the hepatitis-related symptoms of ALT level and HBV DNA copy number decreased, the CCL5 concentration in the patient serum also decreased (Figure 1E).

**Figure 2:**
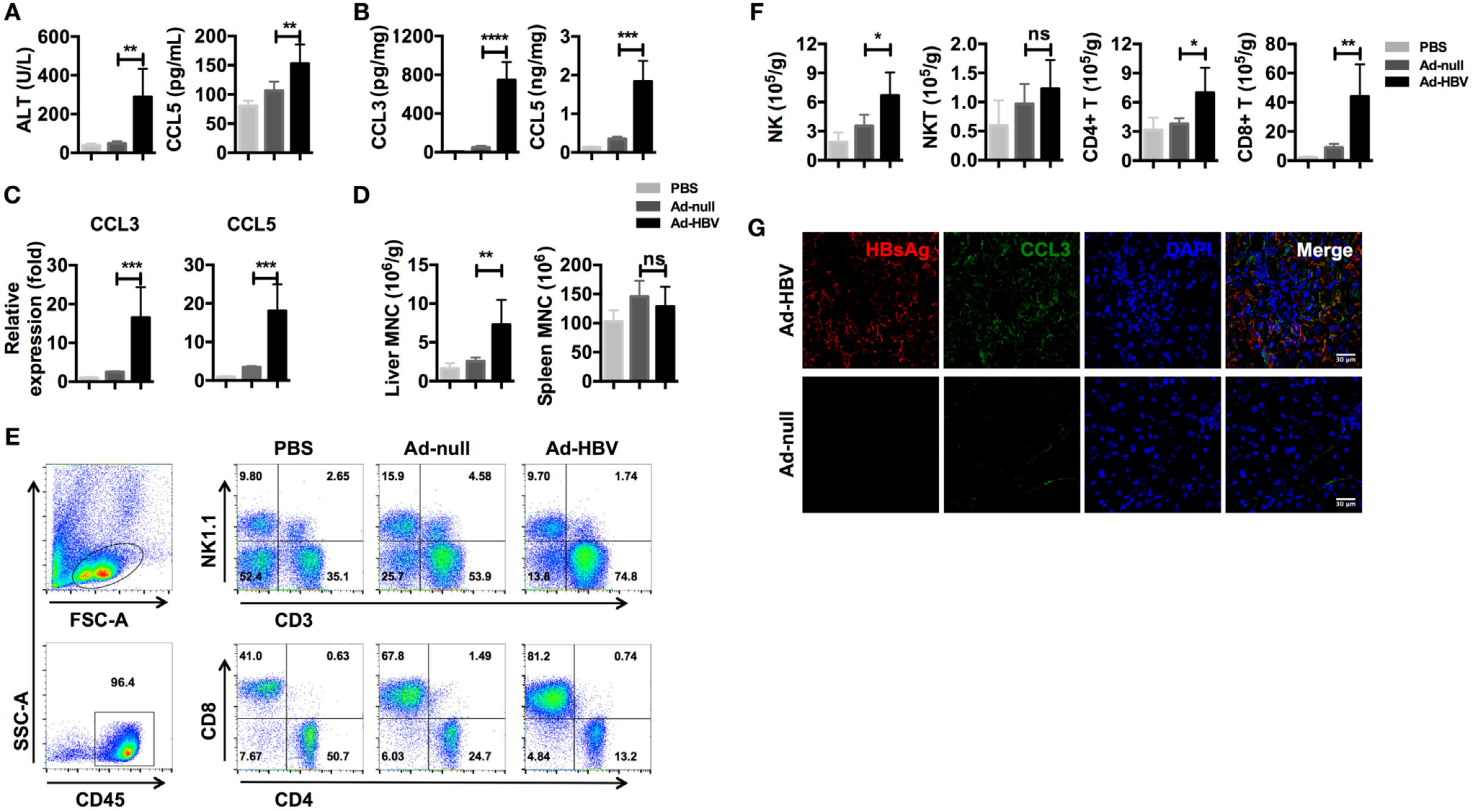
**Ad-HBV infection induces the secretion of inflammation-associated chemokines in the liver**. **(A–G)** C57BL/6 mice were injected i.v. with 1 × 10^9^ pfu Ad-HBV/200 μl PBS (Ad-HBV group) or 1 × 10^9^ pfu Ad-null/200 μl PBS (Ad-null group). About 200 μl of PBS was injected as a normal control (PBS group). Six mice were included in each group. Six days post-injection, the mice were sacrificed for the analysis. **(A)** Serum ALT and CCL5 levels in the mice. **(B)** CCL3 and CCL5 levels in the mouse livers. **(C)** CCL3 and CCL5 mRNA expression levels in the mice. **(D–F)** Mouse livers were harvested for weighing and mononuclear cell isolation. The numbers of total mononuclear cells in the livers and spleens were counted **(D)**. Then, the lymphocyte subtypes were analyzed by flow cytometry **(E)**. The number of cells of each lymphocyte subtype was calculated **(F)**. Frozen sections of mouse livers were stained for HBsAg and CCL3 using immunofluorescence. Scale bars: 30 μm **(G)**.

We used an Ad-HBV infection model of acute viral hepatitis in C57BL/6 mice (41) to determine whether inflammation-associated chemokines were present at higher levels during HBV-mediated hepatitis. As previously reported, mice infected with Ad-HBV showed a high level of serum ALT, which indicated severe liver damage. Meanwhile, the serum CCL5 level was significantly increased in Ad-HBV infection group (Figure 2A). When we examined the expression of CCL3 and CCL5 in the liver, both protein secretion and mRNA expression increased dramatically (Figures 2B,C). CCL3 is another ligand for CCR5, and because CCL3 and CCL5 possess strong chemotactic activity for CCR5+ circulating cells, a large number of immune cells infiltrated the liver in response to the increased expression of these chemokines (Figure 2D). In comparison, mononuclear cells in the spleen exhibited no changes. Analysis of the subtypes of infiltrating lymphocytes revealed a major population of CD8+ T cells (Figure 2E), which tended to be HBV specific and constituted the main cause of hepatitis, according to a previous publication (41). In addition, the accumulation of NK and CD4+ T cells was also substantial, although not as large as that of CD8+ T cells (Figure 2F).

We next investigated chemokine and immune cell localization using frozen liver sections. As expected, the livers of Ad-HBV-infected mice showed robust lymphocyte infiltration, and the hepatocytes with HBV replication secreted a large amount of CCL3 (Figure 2G). These data reveal that CCL3 and CCL5 are hepatitis-associated immune factors.

### HBV DNA Transfection Upregulates CCL3 and CCL5 Expression in Liver-Derived Cells

**Figure 3:**
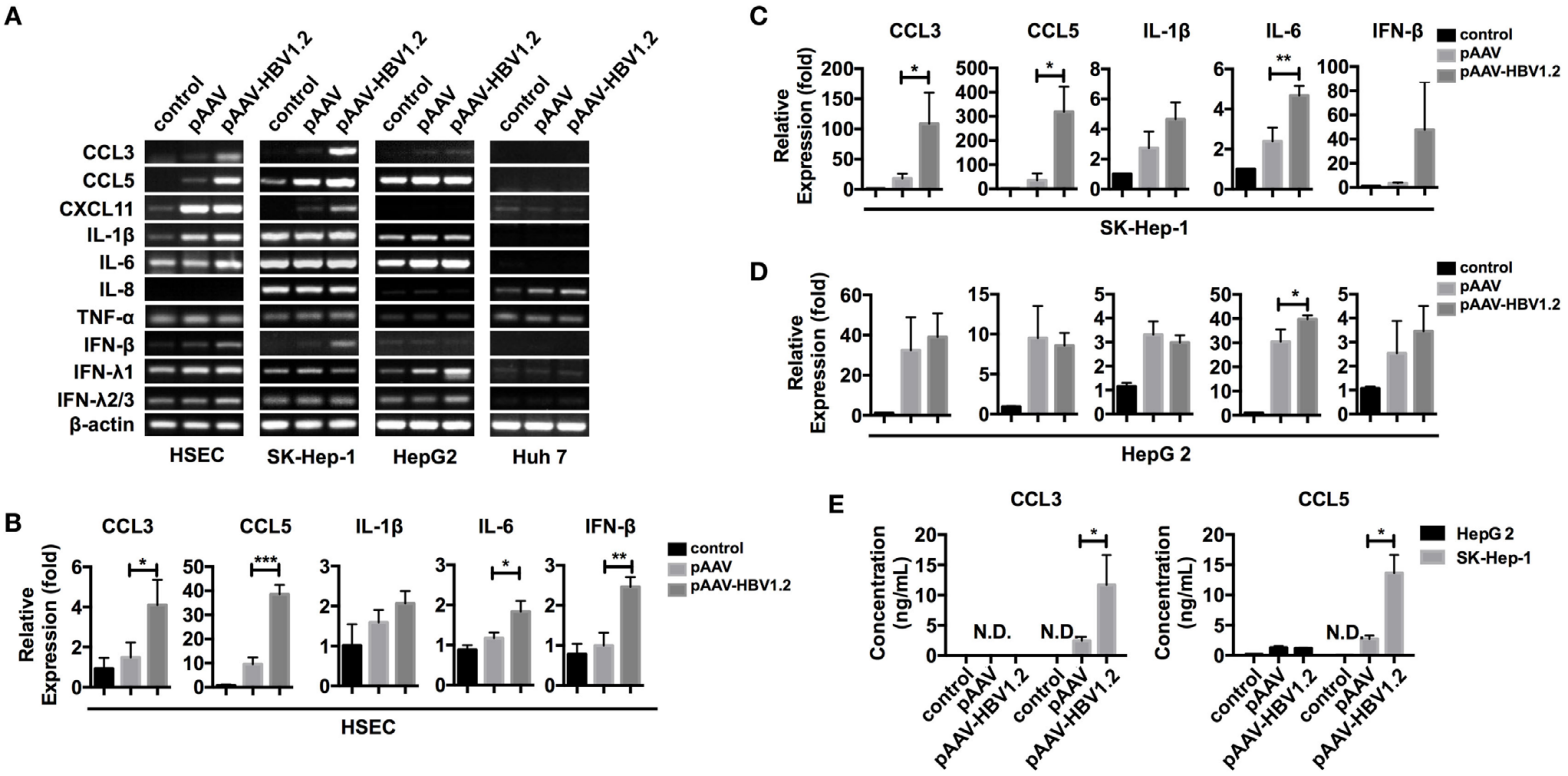
**CCL3 and CCL5 expression increases in liver-derived cells upon HBV plasmid transfection**. **(A–E)** The pAAV-HBV1.2 or pAAV plasmid (1 μg/ml) was transfected into liver-derived cells (0.2 μg/ml for HSECs). Transfection reagent was only applied as a control. RNA isolation and reverse transcription proceeded 48 h post-transfection. **(A)** PCR detection of inflammation-associated chemokine and cytokine mRNAs. **(B–D)** Real-time qPCR determination of chemokine and cytokine mRNA expression (*n* = 3). **(E)** ELISA determination of the secreted CCL3 and CCL5 levels in the cell culture supernatant (*n* = 3).

Chemokine expression can be regulated in various ways in different types of cells. However, whether HBV directly stimulates liver cells to express CCL3 and CCL5 remains to be determined. To imitate HBV genomic DNA presentation in the cytoplasm, we transfected the pAAV-HBV1.2 plasmid into liver-derived cells, i.e., SK-Hep-1, HepG 2, Huh 7, and primary HSECs. Forty-eight hours later, we evaluated the changes in mRNA expression encoding several inflammation-associated cytokines (Figure 3A). The PCR bands revealed that the chemokines CCL3 and CCL5 were strongly increased in HSEC and SK-Hep-1 cells, despite other weak changes. Further real-time qPCR showed that, although some other cytokines such as IL-6 were significantly upregulated, the fold changes in CCL3 and CCL5 were much higher (Figures 3B,D). Meanwhile, CCL3 and CCL5 secretion into HBV plasmid-transfected SK-Hep-1 cell supernatants increased robustly (Figure 3E), confirming the results of mRNA level. Notably, IFN-β, a cytokine that is widely involved in cytosolic innate immune responses, showed an increased mRNA level after HBV plasmid transfection (Figures 3A,D). However, IFN-β was barely detectable in the cell culture supernatant (data not shown), which is similar to HBV infection *in vivo*. This transfection model showed that HBV DNA stimulation directly upregulates CCL3 and CCL5 expression in liver-derived cells.

We next constructed two additional types of HBV plasmid containing 1.3 or 2.3 copies of HBV genomic DNA (Figure S2A in Supplementary Material). In this transfection model, the induction of CCL3 and CCL5 did not depend on the HBV DNA insertion length of the plasmids; all three plasmids efficiently stimulated SK-Hep-1 cells (Figure S2B in Supplementary Material). However, chemokine upregulation depended on the stimulant dosage (Figure S3A in Supplementary Material) and stimulation duration (Figure S3B in Supplementary Material), and we chose 1 μg/ml and 48 h as optimal conditions for subsequent experiments.

### The Cytoplasm-Translocated Ku70/80 Complex Recognizes Transfected HBV DNA

**Figure 4:**
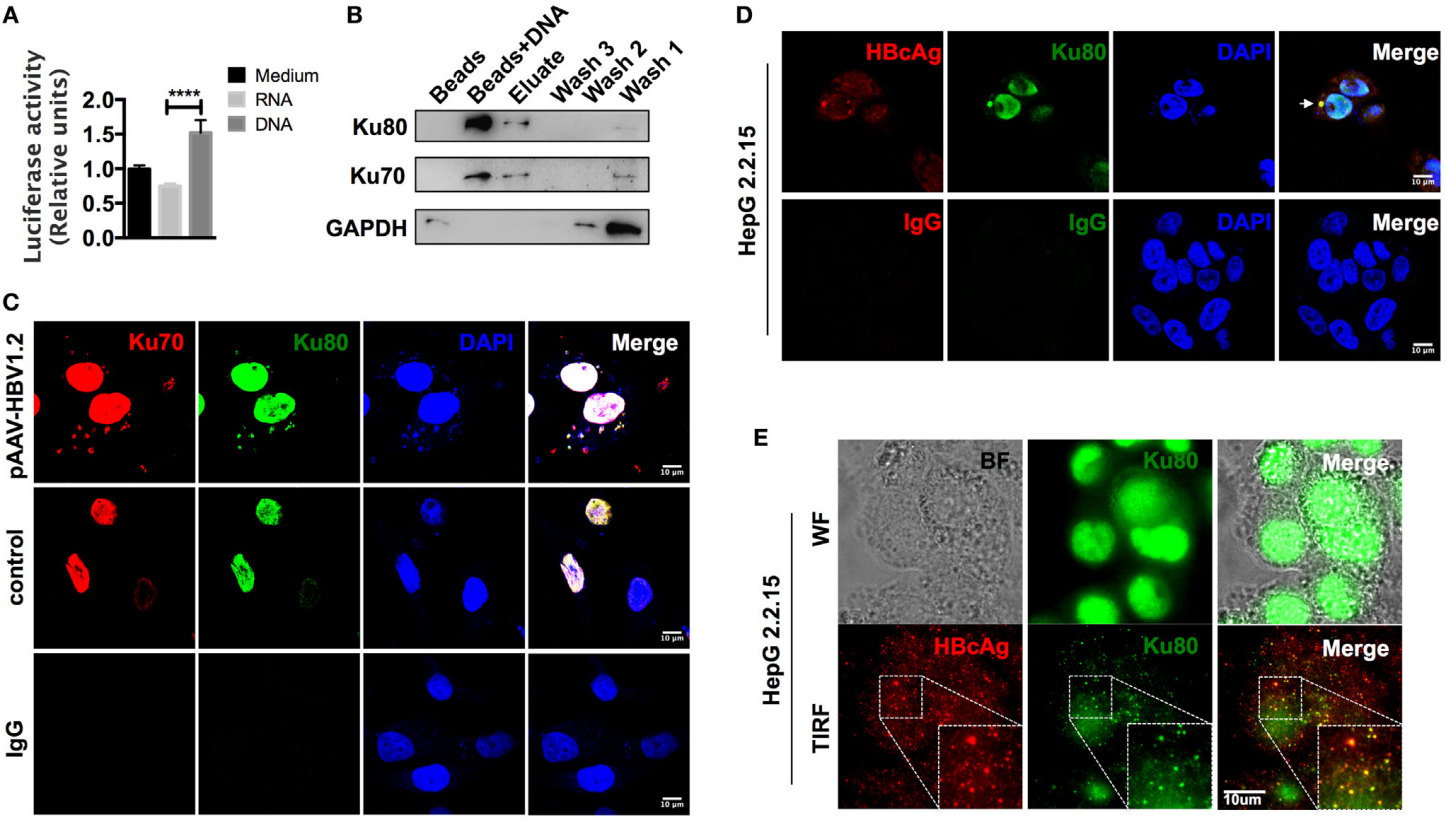
**The cytoplasm-translocated Ku70/80 complex recognizes transfected HBV DNA**. **(A)** Total RNA and cytoplasmic DNA were isolated from HepG 2.2.15 cells. RNA or DNA (1 μg/ml) was transfected into pCCL3p-luc-eGFP-expressing SK-Hep-1 cells. Forty-eight hours later, 150 μg/ml of firefly luciferin was added to the supernatant, and bioluminescence was determined immediately (*n* = 4). **(B)** Biotin-labeled HBV DNA was transfected into SK-Hep-1 cells. Six hours later, the cells were lysed, and the DNA-binding proteins were immunoprecipitated with a μMACS kit. Only beads were used as a control. Three rounds of column wash fluid (lysis buffer, marked Wash1–Wash3) were collected together with the high-salt eluent (1 M NaCl in lysis buffer, marked Elute) and the beads on the column (Marked Beads + DNA). Then, SDS-PAGE was carried out for Western blotting. **(C)** The pAAV-HBV1.2 plasmid was transfected into SK-Hep-1 cells. The cells were then fixed with PFA, and immunofluorescence staining was performed. An equal amount of medium or isotype IgG was applied as a control. Scale bars: 10 μm. **(D,E)** HepG 2.2.15 cells were fixed, and immunofluorescence was assessed. Then, the slides were imaged under confocal **(D)** or wide-field and TIRF modes **(E)**. Scale bars: 10 μm.

Although CCL3 and CCL5 are hepatitis associated, precisely how their expression is regulated remains unclear. Upon observing that cells respond quite rapidly to HBV DNA, within 24 h (Figure S3B in Supplementary Material), and considering that an autocrine feedback mechanism might not be sufficient, we hypothesized that this response was due to direct nucleic acid sensing. As the backbone vector pAAV contained no open reading frames (ORFs) and did not stimulate the cells in comparison to the HBV plasmids (Figure 3; Figure S2 in Supplementary Material), the inserted HBV DNA in the vector was likely the factor that elicited a response. However, it remained unclear whether HBV DNA itself or the transcribed HBV RNAs mediated the observed chemokine upregulation. To address this question, we inserted a luciferase sequence downstream of the CCL3 promoter region and stably expressed the construct in SK-Hep-1 cells. Then, total RNAs and cytosolic DNAs were isolated from HepG 2.2.15 cells, an HBV replication-competent cell line (42), and used for transfection as an HBV nucleic acid stimulator. Although the relationship between HBV RNAs and RIG-I has been identified (17), our data comparing the relative luciferase activity indicated that HBV DNA, rather than RNA, functioned more efficiently in the chemokine upregulation response (Figure 4A).

We next labeled HBV DNAs with 5′-biotin, transfected them into SK-Hep-1 cells, and then pulled down the DNA-binding proteins 6 h later. Through subsequent mass spectrometry analysis (Table S2 in Supplementary Material), we found that the most abundant HBV DNA-binding proteins were the Ku70/80 complex. The Western blot results also confirmed this observation (Figure 4B). Classically responsible for DNA repair reactions, the Ku70/80 complex is mainly localized into the nucleus. However, most of the HBV DNAs were cytosolic 6 h after transfection. To directly examine the localization of the sensing reaction in the cells, we labeled the Ku70/80 complex by immunofluorescence. The confocal images revealed that without transfection stimulation, almost all of the complex molecules remained in the nucleus, whereas a substantial amount was transferred to the cytoplasm and colocalized with HBV DNAs 6 h after transfection (Figure 4C). Moreover, in HepG 2.2.15 cells, we also observed colocalization of Ku80 and HBcAg in the cytoplasm (Figure 4D). With total internal reflection fluorescence (TIRF) microscopy, colocalization of the HBcAg and Ku70 proteins was also visualized near the cell membrane (Figure 4E). Because HBV genomic DNA is reverse transcribed within the assembling core particle during replication (16), this colocalization suggests that HBV DNA could be recognized by the Ku70/80 complex in the cytoplasm.

**Figure 5:**
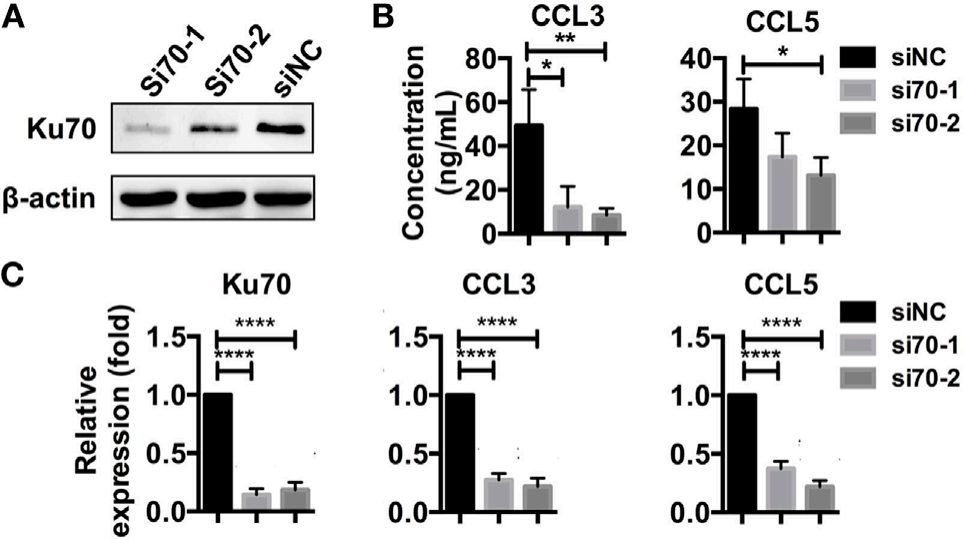
**Knockdown of Ku70 expression impacts chemokine upregulation**. **(A)** SK-Hep-1 cells were treated with Ku70 RNAi. Six hours later, the cells were harvested, and Western blotting was performed to measure Ku70 expression. **(B,C)** SK-Hep-1 cells were treated with RNAi against Ku70 24 h before pAAV-HBV1.2 plasmid transfection. Forty-eight hours later, the secreted CCL3 and CCL5 protein levels in the cell culture supernatant were determined by ELISA **(B)**, and RT-PCR and real-time qPCR were performed to determine the mRNA expression **(C)** (*n* = 3).

Next, we investigated whether HBV DNA sensing by the Ku70/80 complex resulted in chemokine upregulation signals. We used RNA interference (RNAi) targeting Ku70 in SK-Hep-1 cells (Figure 5A) and re-transfected the cells with HBV plasmid. Both the mRNA synthesis and protein secretion of CCL3 and CCL5 were reduced as a consequence of Ku70 knockdown (Figures 5B,C). These data show that HBV DNA sensing by the Ku70/80 complex leads directly to CCL3 and CCL5 upregulation.

### Ku70/80 Drives DNA-PKcs and PARP1 to Promote CCL3 and CCL5 Expression

**Figure 6:**
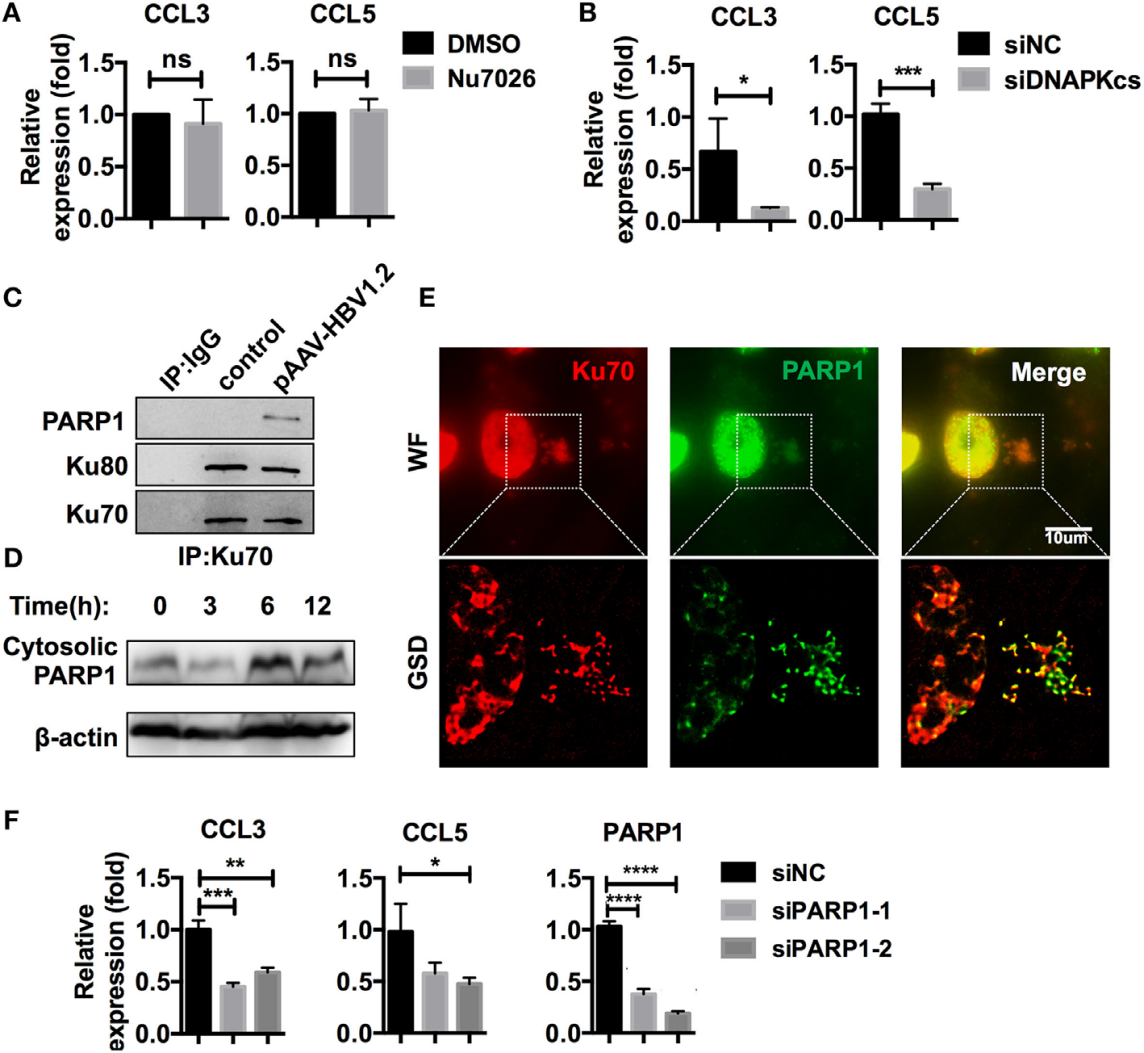
**Ku70/80 elicits DNA-PKcs and PARP-1 to promote CCL3 and CCL5 expression**. **(A)** SK-Hep-1 cells were pre-treated with Nu7026 (10 μM, DMSO as a control) 24 h before transfection with pAAV-HBV1.2 plasmid. Forty-eight hours post-transfection, the cells were harvested using RT-PCR and real-time qPCR assay (*n* = 3). **(B)** SK-Hep-1 cells were treated with RNAi against DNA-PKcs 24 h before pAAV-HBV1.2 plasmid transfection. Forty-eight hours post-transfection, the cells were harvested using RT-PCR and real-time qPCR assays (*n* = 3). **(C)** SK-Hep-1 cells were transfected with pAAV-HBV1.2 plasmid (medium as control). Six hours later, Ku70 was immunoprecipitated with antibody, with IgG1 as a negative control. The samples were analyzed by Western blotting. **(D)** pAAV-HBV1.2 plasmid was transfected into SK-Hep-1 cells. The cells were harvested 0–24 h post-transfection, and the cytosolic proteins were extracted using Western blot. **(E)** SK-Hep-1 cells were transfected with pAAV-HBV1.2 plasmid. Six hours later, the cells were fixed and immunofluorescently labeled. Subsequently, the cells were imaged under wide-field and GSD super-resolution modes. Scale bars: 10 μm. **(F)** SK-Hep-1 cells were treated with Ku70 RNAi 24 h before transfection of pAAV-HBV1.2 plasmid. Subsequently, RT-PCR and real-time qPCR were performed to determine the mRNA expression changes (*n* = 3).

As mentioned earlier, the Ku70/80 complex is known for its DNA repair activities, which also require DNA-PKcs (28). Recently, a DNA-sensing function was reported for Ku70/80, in which DNA-PKcs is involved, independently of its kinase activity (32). In our research, DNA-PKcs also co-precipitated with Ku70 after HBV DNA transfection (Table S3 in Supplementary Material). DNA-PKcs participated in CCL3 and CCL5 regulation in a kinase activity-independent manner because inhibition of its kinase activity by Nu7026 had no effect on subsequent CCL3 and CCL5 upregulation (Figure 6A), while knockdown of DNA-PKcs inhibited this response (Figure 6B). Another DNA repair-related molecule, PARP1, was also a Ku70-co-precipitated protein (Table S3 in Supplementary Material; Figure 6C). Consistent with the Ku70/80 complex, PARP1 demonstrated significant cytoplasmic translocation after HBV DNA transfection (Figure 6D). Moreover, the colocalization of PARP1 and Ku70 was observed by super-resolution microscopy (Figure 6E). When we knocked down PARP1 in SK-Hep-1 cells, the upregulation of CCL3 and CCL5 in response to HBV DNA transfection was affected significantly (Figure 6F). Thus, we believe that the Ku70/80 complex elicits both DNA-PKcs and PARP1 to activate the intracellular signaling cascade that upregulates the chemokines CCL3 and CCL5.

Because there are different DNA-sensing pathways in the cytoplasm, we investigated whether Ku70/80 sensing interacts with multiple DNA-sensing pathways. In addition to inflammasome-associated DNA-sensing pathways, which usually result in IL-1β and/or IL-18 production, the RNA polymerase III/RIG-I and cGAS-STING pathways are crucial methods of cytosolic DNA sensing (13). For this reason, we investigated whether members of these two pathways were recruited to the Ku70/80–DNA-sensing complex by immunofluorescence. However, confocal images showed neither RNA polymerase III nor cGAS colocalized with cytosolic Ku70 proteins (Figure S4A in Supplementary Material), which ruled out the possibility of their direct participation in the HBV DNA response. Because STING functions as a DNA-sensing adapter capable of activating both the IRF3 and NF-κB pathways, on which many cytosolic nucleic acid-sensing pathways rely, we examined the behavior of the STING protein post-transfection. The results suggested that STING did not assemble to the extent of activation and that IRF3 protein nuclear transfer did not occur (Figure S4B in Supplementary Material). Moreover, native PAGE and Western blotting showed that IRF3 proteins remained as inactive monomers (Figure S4C in Supplementary Material). Furthermore, we found that inhibition of the NF-κB pathway did not affect the chemokine upregulation signal (Figure S4D in Supplementary Material). These results indicate no direct roles for typical DNA-sensing pathways in Ku70/80 DNA sensing.

### IRF1 Acts as the Key Transcriptional Factor That Elicits CCL3 and CCL5 Expression

**Figure 7:**
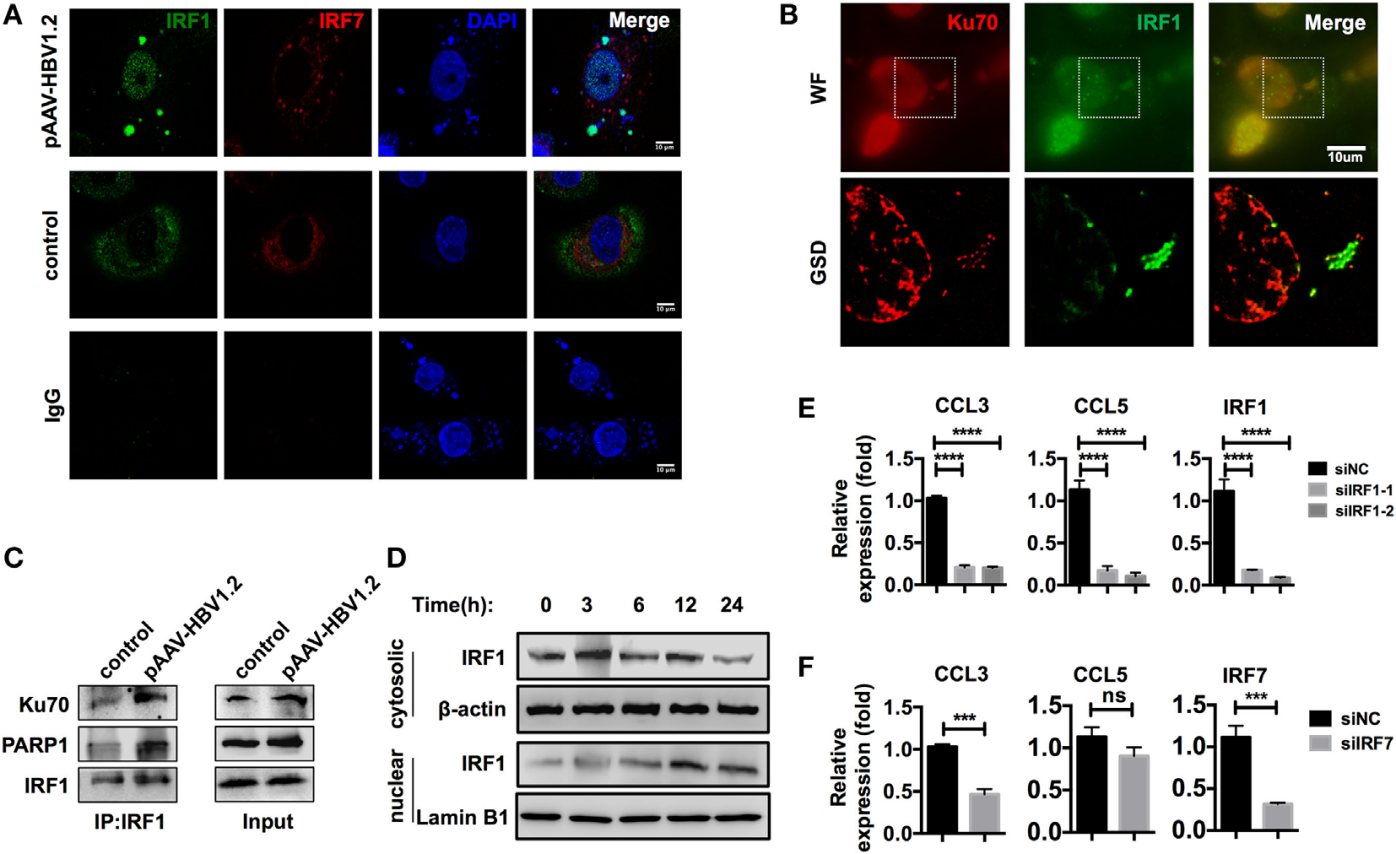
**IRF1 acts as the key transcription factor in this chemokine upregulation pathway**. **(A,B)** SK-Hep-1 cells were transfected with pAAV-HBV1.2 plasmid (medium as control). Six hours later, the cells were fixed and labeled using immunofluorescence. Subsequently, the cells were imaged using confocal **(A)** or GSD systems **(B)** Scale bars: 10 μm. **(C)** SK-Hep-1 cells were transfected with pAAV-HBV1.2 plasmid (medium as control). Six hours later, IRF1 was immunoprecipitated from the cell lysate. Western blotting was performed to analyze the Co-IP samples. **(D)** SK-Hep-1 cells were harvested at 3–24 h after pAAV-HBV1.2 plasmid transfection. IRF1 expression in the cytosol and nuclear was determined by Western blotting. **(E,F)** IRF1 **(E)** or IRF7 **(F)** expression was knocked down by RNAi in SK-Hep-1 cells 24 h before the transfection of pAAV-HBV1.2 plasmid. RT-PCR and real-time qPCR were subsequently used to determine the mRNA expression changes (*n* = 3).

Immunofluorescence data from a previous publication clearly revealed the translocation of IRF3 to the nucleus after stimulation with DNA (32). However, in our model, IRF3 did not seem to be crucial, as it demonstrated no significant nuclear translocation after HBV plasmid transfection (Figures S4B,C in Supplementary Material). In another study, IRF1 and IRF7 were identified as the key transcription factors for activating type III interferon expression (31). Thus, we examined whether these transcription factors played a role in the Ku70/80-mediated chemokine upregulation response. We observed that IRF1 was assembled to DNA sites in the cytoplasm, and nuclear translocation occurred 6 h after transfection, while IRF7 remained cytosolic (Figure 7A). Moreover, in GSD super-resolution images, cytosolic colocalization of Ku70 and IRF1 was observed upon HBV plasmid stimulation (Figure 7B), which implied a direct interaction. To confirm this result, we immunoprecipitated IRF1 and found increased binding of IRF1 to Ku70/PARP1 (Figure 7C). Meanwhile, the IRF1 protein abundance decreased significantly in the cytoplasm, compared to a moderate increase observed in the nuclear (Figure 7D). This response clearly indicated the activation and nuclear transfer of IRF1. Next, we knocked down IRF1 in SK-Hep-1 cells and then stimulated the cells with HBV DNA. As expected, CCL3 and CCL5 mRNA expression decreased dramatically (Figure 7E). In contrast, knockdown of IRF7 decreased only CCL3 expression (Figure 7F). From the above results, we can conclude that IRF1 plays a crucial role as a key transcription factor in the Ku70/80 sensing-mediated chemokine upregulation pathway.

## Discussion

Previous publications suggest an essential role for chemokines in lymphocyte recruitment during hepatitis progression (3, 43). Chemokine secretion is commonly regarded as the consequence of interferon-mediated stimulation. However, HBV infection inhibits type I IFN production by host cells through HBV protein X (44). Here, we report a means of direct regulation of the chemokines CCL3 and CCL5 that are mediated by Ku70/80 sensing of HBV DNA, and this mechanism is the missing link between HBV infection and lymphocyte recruitment. It has been reported that CCL5 expression is reduced in the serum of chronically HBV-infected patients (45). The cohort in that previous study had a normal serum ALT level, indicating mild liver inflammation. However, in our study, all patients had high serum ALT levels, suggesting severe hepatitis. Thus, both results suggest that CCL5 is hepatitis associated. Moreover, our data show that the expression of CCL3 and CCL5 would be upregulated concomitantly in both acute phase and chronic phase patients with high ALT levels. When liver inflammation was relieved using antiviral treatment, the levels of chemokines were downregulated in both responders and non-responders (data not shown). These results suggested that these chemokines are closely related to liver inflammation and not to the other symptoms of hepatitis B. CCR5, as the main receptor for CCL3 and CCL5, is expressed on various immune cells, including NK, NKT, CD4+ T, and CD8+ T cells, and its expression on the cell surface varies in response to HBV infection (27). Thus, the crosstalk between CCR5 and its ligands seems critical for the progression of hepatitis B.

Our experiments used an HBV DNA transfection model in SK-Hep-1 cells, an endothelium-derived liver cancer cell line (46), because these cells exhibit a similar chemokine upregulation response to that of primary HSECs (Figure 3). Although HBV is the prototype of the hepadnavirus family, whose primary host is the hepatocyte, HSECs are also capable of HBV particle uptake (47), which means that it is possible that HBV DNA sensing occurs in these cells. In addition to producing chemokines themselves, HSECs also possess chemokine transfer functions (48). As the major route by which circulating immune cells enter the liver (43, 49), HSEC-mediated increases in chemokine levels are an indication of liver inflammation and mediate the efficient recruitment of lymphocytes.

The amount of HBV DNA delivered to the cells was a crucial experimental parameter for the DNA-binding experiments. Consequently, we used the same concentration of labeled HBV DNA as the plasmid in the HBV plasmid stimulation experiments (1 μg/ml) when performing the transfection. Therefore, the binding activity would occur intracellularly, similar to the HBV plasmid stimulation experiments, which avoided many non-specific protein interactions. Notably, the quantity of DNA that we introduced into cells is much higher than that in HBV-infected cells. Therefore, the amount of HBV DNA level used for mass spectrometry identification of DNA-binding protein might not match that of natural HBV infection. Nevertheless, this kind of DNA transfection model was proved to be valid in DNA sensor identification by previous researches such as the finding of IFI16 sensing HIV cDNA in CD4+ T cells (22). Moreover, we observed colocalized HBcAg and Ku80 in the cytoplasm of HepG2.2.15 cells (Figures 4D,E), which suggested natural binding of Ku proteins to HBV DNA. Our data also showed dose-dependent HBV DNA-mediated chemokine upregulation. Thus, the first wave of CCL3 and CCL5 chemokine secretion *in vivo* may be subtle, but it could still be capable of recruiting immune cells. The recruited cells early in infection interact with the infection microenvironment, leading to more cytokine secretion and more immune cell recruitment. Indeed, CCL3 and CCL5 upregulation mediated by HBV DNA sensing by the Ku70/80 complex may initiate this type of cascade reaction.

As Ku70 and Ku80 are DNA repair-associated proteins, the Ku70/80 complex is predominantly localized to the nucleus, whereas DNA sensing primarily occurs in the cytosol. How this translocation is precisely achieved remains unclear. One hypothesis focuses on the dynamic equilibrium between nuclear and cytosolic Ku proteins. Cytosolic Ku proteins may bind to and assemble on exogenous DNAs upon viral infection, which would disrupt the equilibrium and drive Ku proteins from the nucleus. Our Ku70 Co-IP MS data (Table S3 in Supplementary Material) also suggest the binding of myosin and plectin, indicating an unknown mechanism of Ku protein transfer. Considering the similar DNA-binding role and similar subsequent molecules that they recruit within both DNA repair and cytosolic DNA-sensing reactions, Ku proteins seem to recognize DNA with no bias. Nevertheless, the location of the recognition should indicate the type of reaction. Molecules used for DNA repair are abundant in the nucleus; thus, Ku proteins enter into DNA repair pathways without hindrance. However, in the cytosol, which is usually a DNA-free environment, molecules needed for DNA repair are rarely accessible. Instead, DNA-sensing pathways are much easier to deploy. Through an as-yet-undetermined mechanism, this results in increased IFN-λ and/or chemokine expression.

The Ku70/80 complex has been shown to be capable of sensing all types of DNA, with a preference for ssDNA and dsDNA with strand breaks (31), which is consistent with its DNA repair function. From minus-strand reverse transcription to relaxed circular DNA (rcDNA), HBV DNA in the cytoplasm of host cells always remains non-cyclic, which is consistent with the characteristics of Ku proteins. Notably, in the previous publication (22), the authors focused on the sensing function of IFI16. However, their data also demonstrated that the highest-ranked proteins on the list of HIV DNA-binding proteins that were immunoprecipitated were Ku70/80 and PARP1. This may suggest that Ku70/80 elicits a common response upon sensing cytosolic DNA during infection by viruses that use a reverse transcription replication strategy.

## Author Contributions

YoL performed experiments. YW, XZ, and JC did some experiments. YaL and JL communicated with HBV patients and collected the patient serum, testing ALT, and HBV DNA. RS established techniques of flow cytometry and interpreted the data, ZT and HW designed the study, supervised research, and revised the manuscript.

## Conflict of Interest Statement

The authors declare that the research was conducted in the absence of any commercial or financial relationships that could be construed as a potential conflict of interest. The reviewer BN and handling Editor declared their shared affiliation, and the handling Editor states that the process nevertheless met the standards of a fair and objective review.
