# Supplementary material for: Cytoplasm-Translocated Ku70/80 Complex Sensing of HBV DNA Induces Hepatitis-Associated Chemokine Secretion
Source: Front Immunol. 2016 Dec 5;7:569. doi: 10.3389/fimmu.2016.00569 (PMC5136554; doi:10.3389/fimmu.2016.00569)
Supplement: Supplementary file 4 [file Image_1.PDF]

Supplementary Material

Cytoplasm-translocated Ku70/80 complex sensing of HBV DNA induces hepatitis-associated chemokine secretion

Young Li[1,3], Yang Wu[1,3], Xiaohu Zheng[1,3], Jingjing Cong[1,3], Yanyan Liu[4], Jiabin Li[4], R. Sun[1,2,3], Z.G. Tian[1,2,3*], H.M. Wei[1,2,3*]

*** Correspondence:**

H.M. Wei: ustcwhm@ustc.edu.cn
Z.G. Tian: tzg@ustc.edu.cn

Supplementary figure legends

A

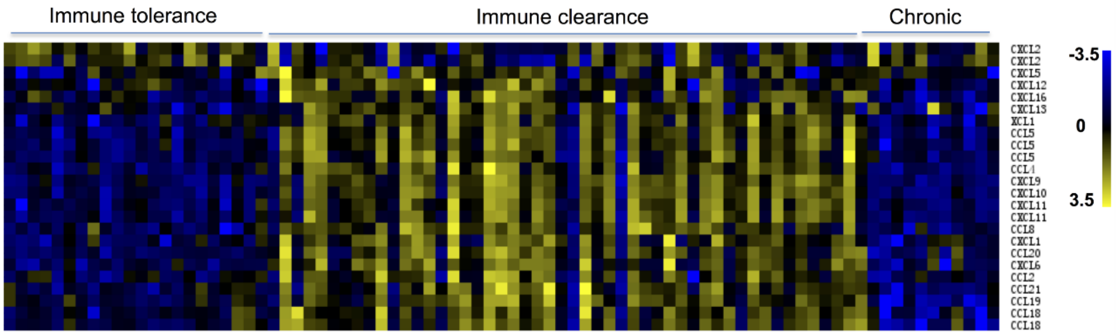

B

Rank	GENE_SYMBOL	Clearance vs Tolerance & Chronic (p value)
1	CXCL10	4.44E-16
2	CXCL9	8.88E-16
3	CCL4	4.57E-14
4	CCL5	2.35E-13
5	CCL5	1.30E-12
6	CXCL11	2.09E-12
7	CXCL11	6.33E-12
8	CCL20	7.70E-12
9	CCL5	4.86E-11
10	CXCL6	8.63E-11

C

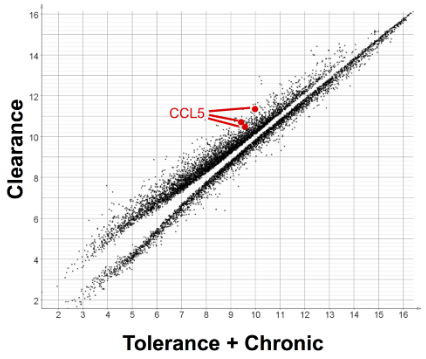

Figure S1. The chemokine CCL5 is highly expressed in hepatitis B patients in the immune clearance phase. (A) Heat map of chemokine expression with Student's t-test p-values less than 0.01 (immune clearance vs. immune tolerance + chronic). The data were derived from GSE65359 and normalized to MeV. (B) Chemokines with top p-value ranks in (A). (C) Scatter plot of genes with p-values less than 0.01 in GSE65359. Three CCL5 probes are indicated.

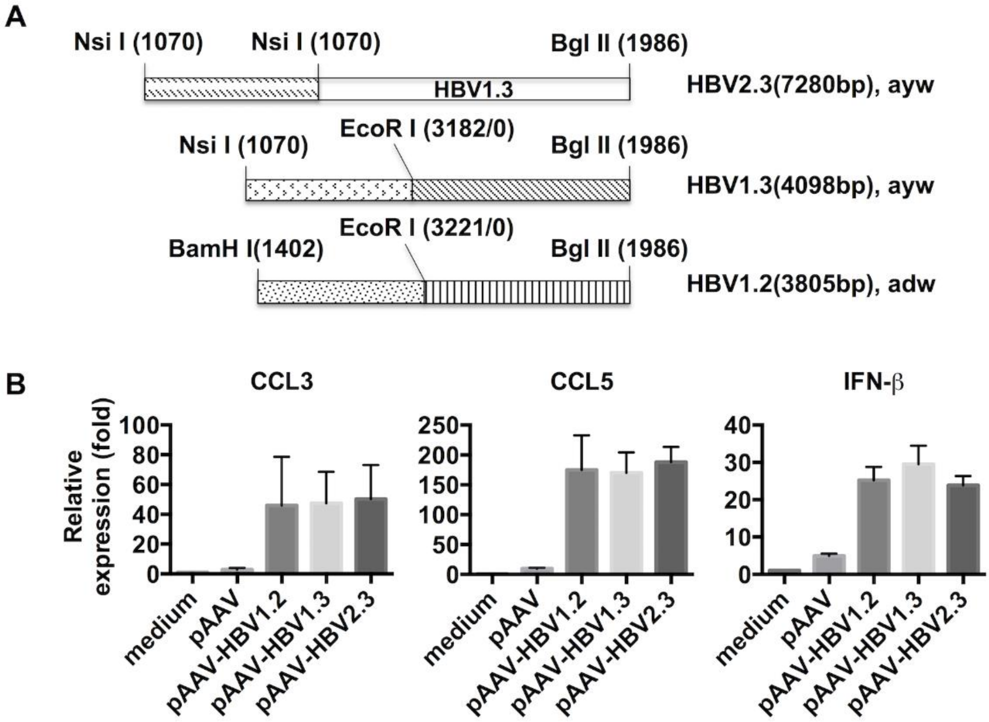

Figure S2. Change of inserted HBV genome sequence length has no affect on stimulation. (A) The structures of the pAAV-HBV1.2, pAAV-HBV1.3, and pAAV-HBV2.3 plasmids. (B) Each plasmid (1 ug/mL) was transfected into SK-Hep-1 cells. An equal amount of pAAV plasmid or medium only was applied as a control. RT-PCR and real-time qPCR were performed 48 h later, n=3.

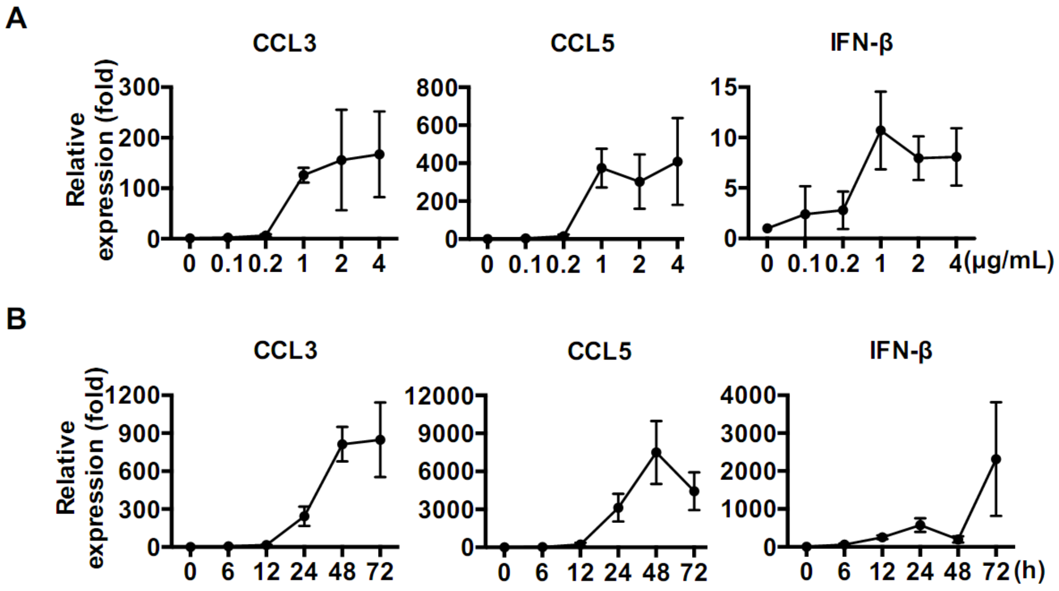

Figure S3. The chemokine upregulation response is stimulation dose- and duration-dependent. (A) A total of 0-4 ug/mL of pAAV-HBV1.2 plasmid was transfected into SK-Hep-1 cells. RT-PCR and real-time qPCR were performed 48 h later, n=3. (B) Each plasmid (1 ug/mL) was transfected into SK-Hep-1 cells. Samples were harvested 6 to 72 h later, as indicated, n=3.

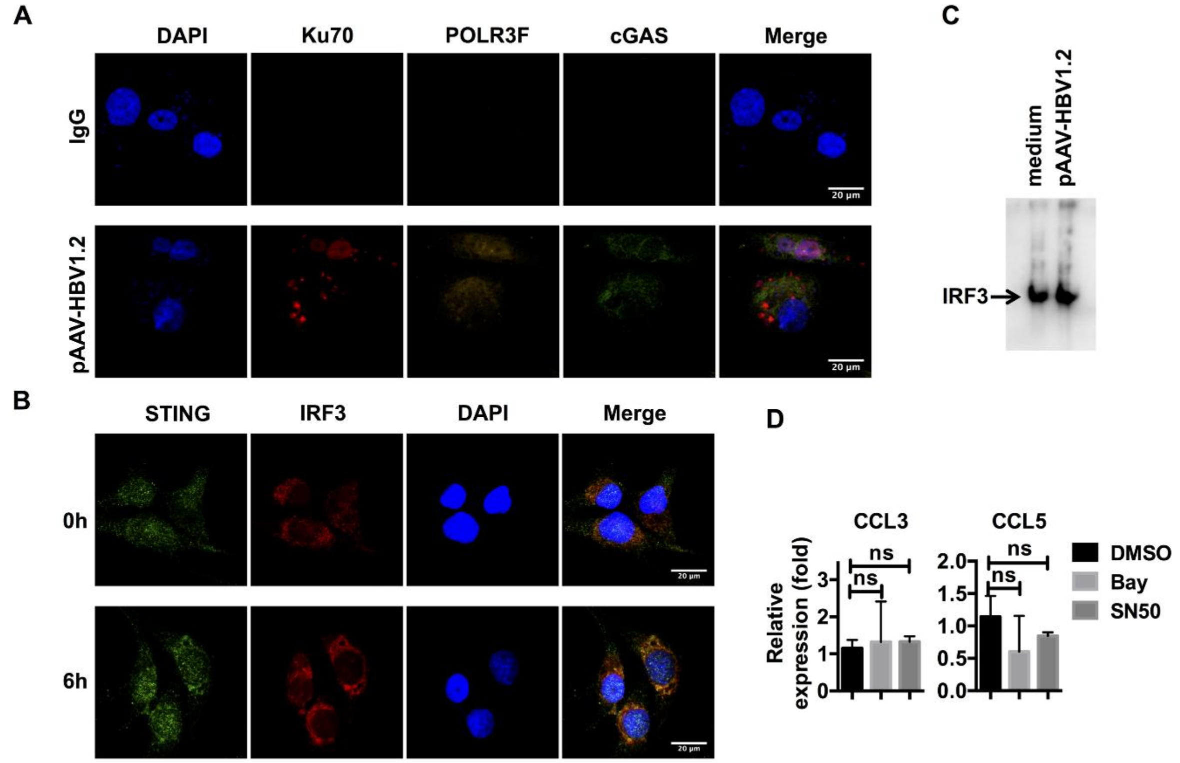

Figure S4. The cGAS-STING-IRF3 axis is not involved in the sensing of transfected HBV DNA. (A, B) The pAAV-HBV1.2 plasmid was transfected into SK-Hep-1 cells. Six hours post-transfection, the cells were fixed with 4% PFA, and immunofluorescence staining was performed. An equal amount of medium or isotype IgG was used as a control. (C) pAAV-HBV1.2 plasmid was transfected into SK-Hep-1 cells. Six hours post-transfection, the cells were lysed with NP-40 lysis buffer, and native page and western blot detection of IRF3 were performed. (D) SK-Hep-1 cells were pre-treated with Bay-11-7028 (5 μM) or SN50 (50 μM) for 24 h, followed by pAAV-HBV1.2 plasmid transfection. Forty-eight hours post-transfection, the cells were harvested for RT-PCR and real-time qPCR assays. An equal volume of DMSO was used as a negative control.

Supplemental Table 1. Antibodies, siRNA and PCR primer sequences used and mass spectrometry results.

Supplemental Table 2. MS data of HBV DNA-binding proteins.

Supplemental Table 3. MS data of Ku70 co-IP samples.
